# Stressor controllability modulates the stress response in fish

**DOI:** 10.1186/s12868-021-00653-0

**Published:** 2021-08-04

**Authors:** Marco Cerqueira, Sandie Millot, Tomé Silva, Ana S. Félix, Maria Filipa Castanheira, Sonia Rey, Simon MacKenzie, Gonçalo A. Oliveira, Catarina C. V. Oliveira, Rui F. Oliveira

**Affiliations:** 1 Centro de Ciências Do Mar (CCMAR), Universidade Do Algarve, 8005-139 Faro, Portugal; 2 Fish Welfare Initiative, Normal, IL 61761 USA; 3 Laboratoire Ressources Halieutiques, Ifremer, 17137 L’Houmeau, France; 4 SPAROS, 8700-221 Olhão, Portugal; 5 ISPA—Instituto Universitário, 1149-041 Lisbon, Portugal; 6 Instituto Gulbenkian de Ciência, 2780-156 Oeiras, Portugal; 7 Institute of Aquaculture, University of Stirling, Stirling, FK9 4LA UK; 8 Champalimaud Research, 1400-038 Lisbon, Portugal

**Keywords:** Stress, Controllability, Cortisol, Immediate early genes, Dorsolateral pallium, Fish welfare

## Abstract

**Background:**

In humans the stress response is known to be modulated to a great extent by psychological factors, particularly by the predictability and the perceived control that the subject has of the stressor. This psychological dimension of the stress response has also been demonstrated in animals phylogenetically closer to humans (i.e. mammals). However, its occurrence in fish, which represent a divergent vertebrate evolutionary lineage from that of mammals, has not been established yet, and, if present, would indicate a deep evolutionary origin of these mechanisms across vertebrates. Moreover, the fact that psychological modulation of stress is implemented in mammals by a brain cortical top-down inhibitory control over subcortical stress-responsive structures, and the absence of a brain cortex in fish, has been used as an argument against the possibility of psychological stress in fish, with implications for the assessment of fish sentience and welfare. Here, we have investigated the occurrence of psychological stress in fish by assessing how stressor controllability modulates the stress response in European seabass (*Dicentrarchus labrax*).

**Results:**

Fish were exposed to either a controllable or an uncontrollable stressor (i.e. possibility or impossibility to escape a signaled stressor). The effect of loss of control (possibility to escape followed by impossibility to escape) was also assessed. Both behavioral and circulating cortisol data indicates that the perception of control reduces the response to the stressor, when compared to the uncontrollable situation. Losing control had the most detrimental effect. The brain activity of the teleost homologues to the sensory cortex (Dld) and hippocampus (Dlv) parallels the uncontrolled and loss of control stressors, respectively, whereas the activity of the lateral septum (Vv) homologue responds in different ways depending on the gene marker of brain activity used.

**Conclusions:**

These results suggest the psychological modulation of the stress response to be evolutionary conserved across vertebrates, despite being implemented by different brain circuits in mammals (pre-frontal cortex) and fish (Dld-Dlv).

**Supplementary Information:**

The online version contains supplementary material available at 10.1186/s12868-021-00653-0.

## Background

It has long been established that psychological factors, such as controllability and predictability, can be as important as intrinsic characteristics of the stressor (e.g. intensity) on the effects of stress in humans [1]. Interestingly, this psychological dimension of the stress response was first investigated in laboratory animals, in particular in monkeys, dogs and rodents (e.g. [2–7]) and only subsequently extended to humans (e.g. [8–10]). The seminal studies of stressor controllability in animal models have compared the stress response in animals that can escape the stressor (i.e. that has control over the stressor, originally a tail-shock) to animals exposed to the same stressor but that have no control over it. The effects of lack of stressor control (aka learned helplessness), include increased anxiety, decreased social exploration, and heightened fear conditioning and delayed fear extinction [1]. These effects are not observed in animals that have controllability over the stressor. Similarly, in humans, laboratory tasks that induce uncontrollable (but not controllable) stress impair fear extinction and executive functioning in a Stroop task [11, 12], and increase perceived helplessness, depression and anger [13, 14].

Research on the neural mechanisms of stressor controllability suggests that the blunted stress response induced by perceived stressor control is due to a corticostriatal circuit involving the ventral medial pre-frontal cortex (mPFC) and the posterior dorsomedial striatum that exerts top-down inhibitory control over subcortical stress-responsive structures, namely the serotonergic system of the dorsal raphe nucleus and the amygdala [15]. At the neuroendocrine level, the hypothalamic–pituitary–adrenal (HPA) axis response to stress has been shown to be influenced by controllability in humans, where in response to laboratory tasks, cortisol levels are lower in groups where stressor control is perceived than in groups where stressor control is not perceived [14] and perceived control is negatively correlated with cortisol [16, 17]. In contrast, in rodents the HPA stress response seems insensitive to stressor controllability, but repeated exposure to controllable stressors leads to a lower response [18–20]. Given the central role of the mPFC on stressor controllability, it could be questioned whether this psychological moderation of the stress response is also present in non-mammals that lack a cerebral cortex. In this respect, querying its occurrence in fish, which represent a phylogenetically divergent vertebrate lineage from that of mammals [21], whose brains lack a mPFC homologue [22], is of major relevance. Its presence in fish would indicate that stressor controllability is an ancestral psychological trait that can be implemented by different neural substrates in fish and mammals. In fact, the computations needed for similar cognitive abilities can be implemented by similar neuronal circuitry irrespective of its organization in a layered or nuclear architecture. This has recently been shown by the involvement of the mesopallium and nidopallium in the complex cognitive abilities of birds [23–25].

In teleost fish three pallial areas and one sub-pallial area have been suggested to play a role in cognitive and affective components of behavior: (1) the medial zone of the dorsal telencephalic area (Dm), which has been shown to be involved in emotional learning [26, 27] and is the putative homologue of the mammalian pallial amygdala [28, 29]; (2) the ventral division of the lateral telencephalic area (Dlv), which has been shown to be involved in spatial and time-related learning [30–32] and is the putative homologue of the mammalian hippocampus [28, 29]; (3) the dorsal division of the lateral telencephalic area (Dld), which has been shown to integrate multimodal sensory information and is the putative homologue of the sensory cortex [28, 29]; and (4) the ventral nucleus of the ventral telencephalic area (Vv), which has been shown to respond to stimulus salience [33] and is a putative homologue of the lateral septum and of the nucleus accumbens [34, 35]. The role of psychological factors in the regulation of the stress response in fish has been seldom addressed [36], and when it has, the focus has predominantly been on the effects of stressor predictability [37–40], in which species-specific neural responses have been reported [[e.g. increased activity in Vv in seabream, [33]; and increased activity in Dm and decreased activity in Dlv in Sea Bass, in which Vv was not sampled, [41]]. To the best of our knowledge, the effect of controllability in fish has been only addressed in a study in which rainbow trout (*Oncorhynchus mykiss*) had the chance to actively avoid being defeated by a larger conspecific in a conditioning paradigm. In this study, the trout that could escape the social stressor exhibited a lower cortisol response to the conditioned stimulus than those that cannot escape social defeat [42]. However, this evidence has not yet been investigated as supporting the occurrence of psychological stress in fish, and its neural bases have not been investigated yet in fish.

Here, we investigated the occurrence of stressor controllability in the European seabass (*Dicentrarchus labrax*), which is a key species for European aquaculture. Thus, the results of this study will not only allow testing the hypothesis of the deep evolutionary origin of stressor controllability effects on the stress response and its brain substrates, but also has direct implications for the welfare of farmed fish. For this purpose, we used a conditioning protocol to associate a conditioned stimulus (CS = light) with a stressor (US = confinement) under different conditions: (1) Controllable stressor (CTR)—fish had the choice to escape from US by a door; (2) Uncontrollable stressor (UnCTR)—fish had no choice to escape from US; and (3) Loss of stressor control (CTRUn)—fish were subjected during 5 conditioning sessions to the same conditions as CTR followed by 2 sessions under UnCTR conditions (Additional file 1: Table S1). In the test session, all experimental groups were exposed to the CS in the absence of the US. We have characterized the effects of controllability on the stress response at multiple levels: behavior, circulating cortisol and activation of the 4 candidate teleost homologues of tetrapod subcortical regions involved in cognitive appraisal of stressors discussed above (i.e. Dm, Dld, Dlv, Vv), using immediate early genes (IEG: *egr-1*, *c-fos*, *bdnf* and *npas4*) as markers of neural activity.

## Results

### Effect of stressor controllability on behavior

**Fig. 1:**
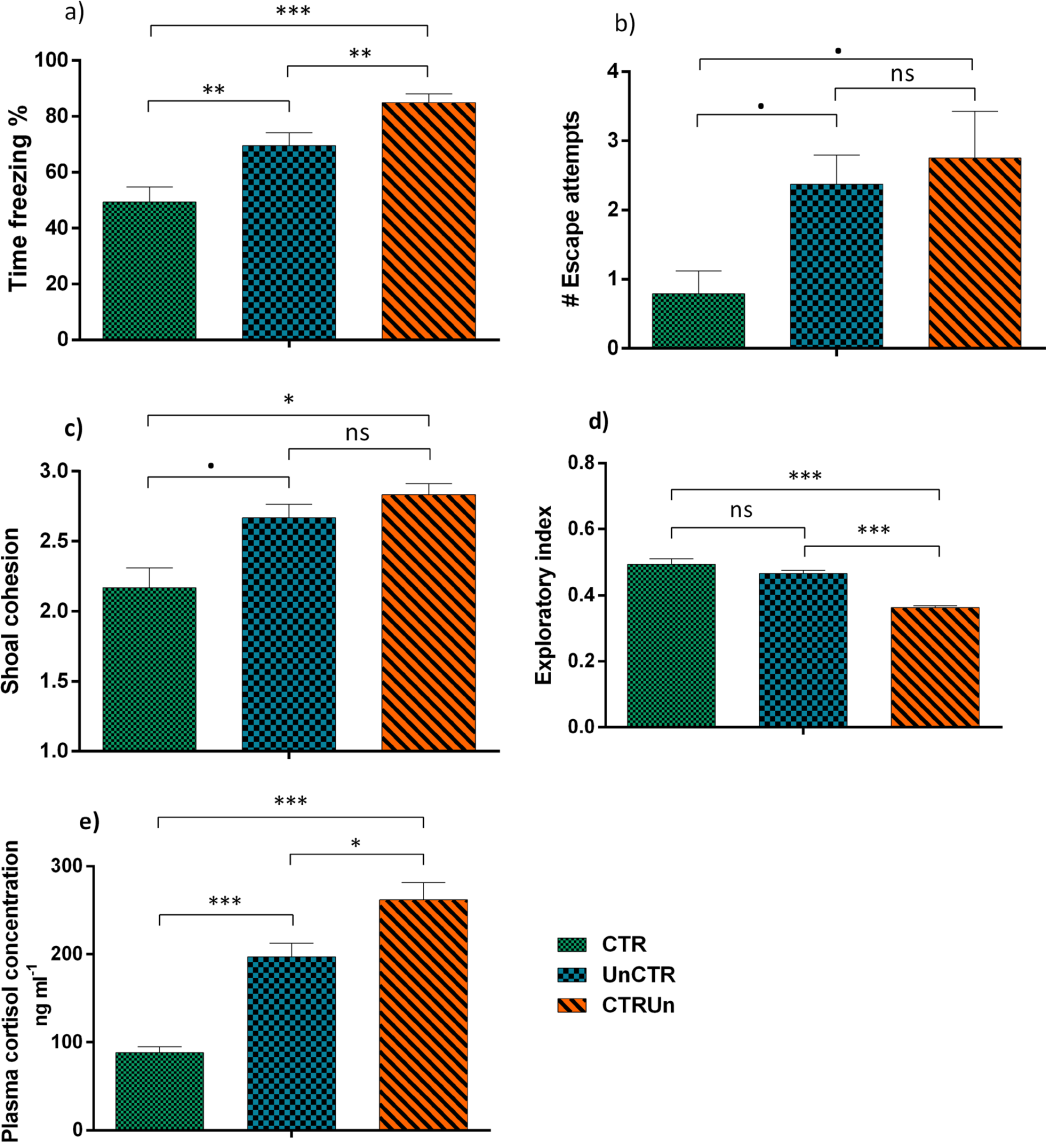
Behavioral and physiological responses of seabass expressed by fish during the test session (experimental treatments: CTR = stressor controllability; UnCTR = stressor uncontrollability; CTRUn = loss of stressor controllability): **a** Time in freezing (%), **b** frequency of escape attempts, **c** shoal cohesion, **d** Exploratory behavior, **f **plasma cortisol concentrations measured 30 min after the test session. Linear mixed models with planned comparisons are indicated; * p < .05; ** p < .01; *** p < .001; ns—non-significant. All descriptive statistics are mean ± SEM

Analysis of the behavior from the test session showed a significant effect of controllability on all behavioral variables measured (Table 1; Fig. 1a–d). Freezing was higher in the loss of control treatment, than in the uncontrolled or controlled treatments, and the latter two treatments also differed significantly (Fig. 1a). The number of escape attempts and shoal cohesion showed a similar pattern with both the uncontrolled and loss of control treatments, showing higher levels that the control treatment (Fig. 1b, c). Conversely, exploration was significantly lower in the loss of control treatment than in any of the other two treatments (Fig. 1d).

**Table 1:** Linear Mixed Model main effects of the behavioral, physiological and of IEGs mRNA responses expressed between experimental conditions (i.e. CTR = stressor controllability; UnCTR = stressor uncontrollability and CTRUn = loss of stressor controllability)

Behaviors	df	Time freezing		Escape events		Shoal cohesion		Exploratory behavior		Cortisol	
		***F***	***p***	***F***	***p***	***F***	***p***	***F***	***p***	***F***	***p***
	2.15	17.55	** < 0.001**	2.88	**0.087**	3.74	**0.048**	13.9	** < 0.001**	45.1	** < 0.001**

Seabass nuclei		Dm		Dld		Dlv		Vv			
IEGs		*F*	*p*	*F*	*p*	*F*	*p*	*F*	*p*		
*egr-1*	2.4	1.07	0.425	1.66	0.297	2.53	0.194	2.66	0.184		
*c-fos*	2.4	0.93	0.465	2.00	0.250	4.653	0.090	0.157	0.859		
*Bdnf*	2.4	1.41	0.344	2.56	0.192	3.74	0.121	6.03	0.062		
*npas4*	2.4	0.93	0.464	2.16	0.230	6.92	0.050*	0.43	0.675		

Time in freezing, frequency of escape events, shoal cohesion, and exploratory behavior expressed during the test session and cortisol levels and immediate early gene (IEGs) mRNA expression measured 30 min after the test session. IEGs mRNA from each of the candidate brain nuclei in seabass (i.e. Dm, Dld, Dlv and Vv) are indicated. Significant differences are expressed with * (p < 0.05)

### Effect of stressor controllability on cortisol

Stressor *c*ontrollability significantly affected cortisol levels (Table 1): control over the stressor decreased cortisol levels, whereas loss of controllability induced higher cortisol levels than uncontrolled stress itself (Fig. 1f).

### Effect of stressor controllability on patterns of brain activation and neurogenomic states

**Fig. 2:**
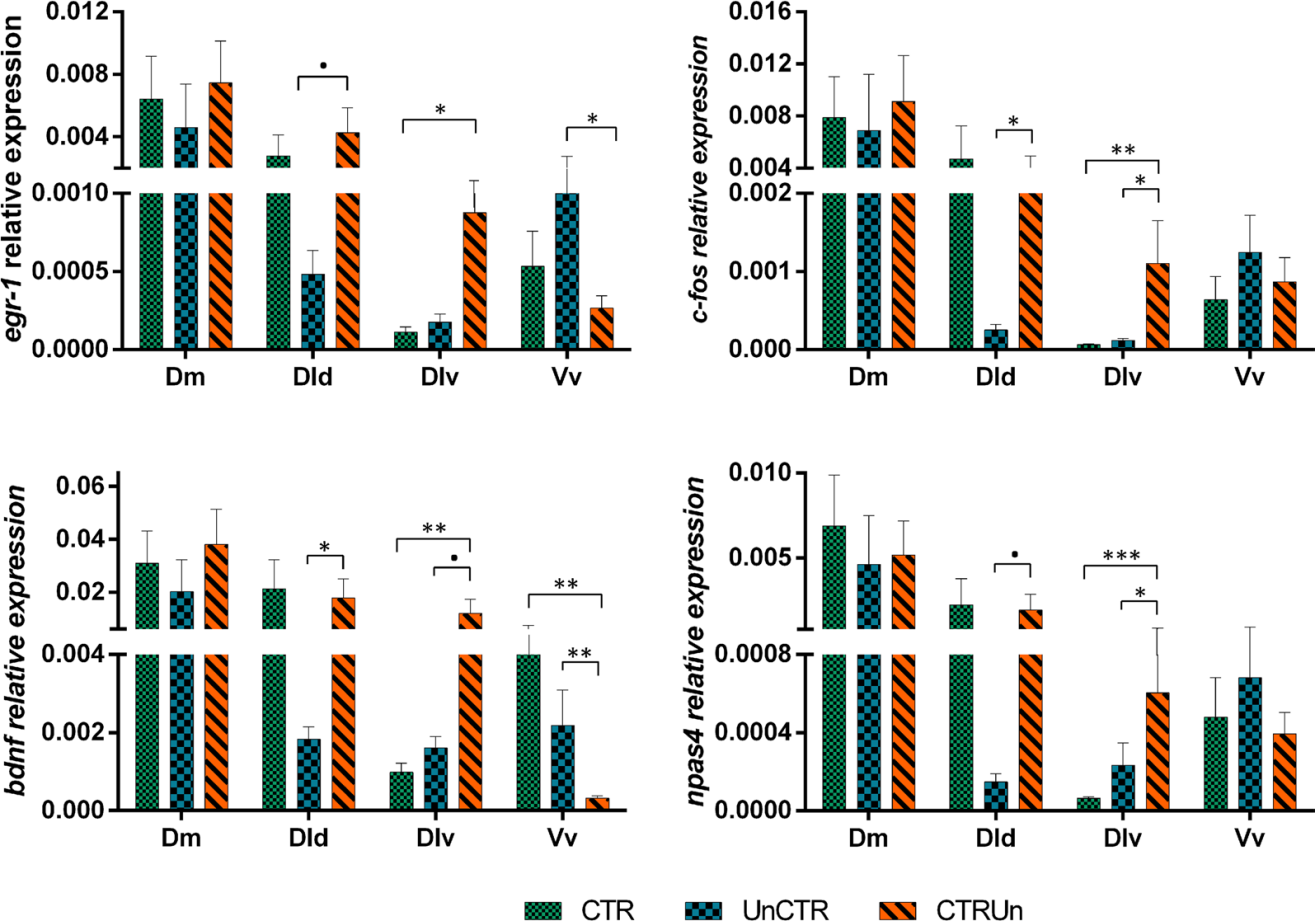
Expression (mean ± SEM) of the immediate early genes *egr-1, c-fos, bdnf* and *npas4* in the Dm, Dld, Dlv and Vv regions of Sea bass brain under different experimental conditions (CTR = stressor controllability; UnCTR = stressor uncontrollability; CTRUn = loss of stressor controllability). Significant differences (planned comparisons) in expression levels between experimental conditions (i.e. CTR vs. UnCTR; CTR vs. CTRUn and UnCTR vs. CTRUn) are indicated by asterisks: * p < .05; ** p < .01; *** p < .001; ns—non-significant

Stressor controllability elicited different patterns of brain activation towards the stressor, as measured by the expression of immediate early genes, used as indicators of brain activity (Table 1; Fig. 2). In the Dlv, the loss of stressor control induced higher activity than either exposure to a controlled stressor (as indicated by the expression of all IEGs), or exposure to an uncontrolled stressor (as indicated by the expression of *c-fos*, *bdnf* and *npas1*). In the Dld, the loss of stressor control induced higher activity than exposure to an uncontrolled, but not to a controlled stressor (as indicated by the expression of all IEGs), with similar levels of IEG expression in controlled and loss of control treatments. In Vv loss of control is associated with lower *egr-1* expression than exposure to an uncontrolled stressor, and the expression of *bdnf* is higher when exposed to a controllable stressor than when exposed to either an uncontrolled stressor or when there is a loss of control of the stressor. Finally, for Dm there were no significant differences in IEG expression between treatments.

**Fig. 3:**
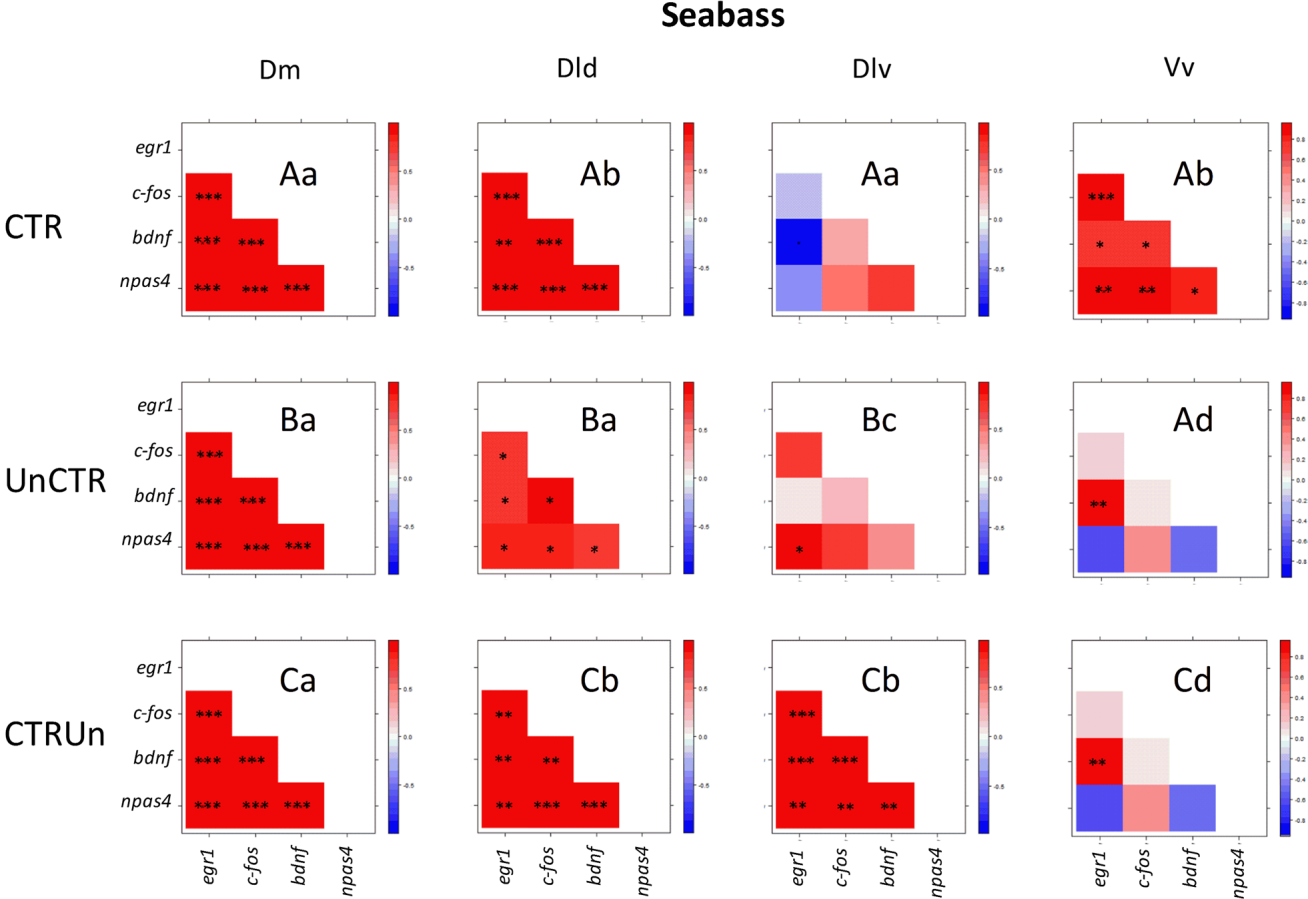
Neurogenomic states of seabass, as described by correlation (r) matrices of immediate early genes expression in the different brain nuclei (Dm, medial zone of the dorsal telencephalic area; Dl, lateral zone of the dorsal telencephalic area; Dld, dorsal lateral zone of the dorsal telencephalic area; Dlv, ventral lateral zone of the dorsal telencephalic area; Vv, ventral nucleus of the ventral telencephalic area) for each experimental treatment (CTR = stressor controllability; UnCTR = stressor uncontrollability; CTRUn = loss of stressor controllability); Color scheme represents r-values from − 1 (blue) to 1 (red); Asterisks indicate significant correlations after p-value adjustment for multiple correlations: *p < 0.05; **p < 0.01; ***p < 0.001; different capital letters indicate significantly different co-expression patterns among experimental treatments, and different small letters indicate significantly different co-expression patterns among brain nuclei, using the QAP correlation test

The neurogenomic states, as characterized by the pattern of co-expression of the immediate early genes on each brain region, are specific for each experimental treatment (i.e. CTR vs. UnCTR vs. CTRUn) in Dm, Dld and Dlv (Fig. 3). The neurogenomic state of Vv is similar between controllable and uncontrolled treatments (see Additional file 1: Table S1 for detailed information on QAP correlations, used to infer significance differences between pairs of co-expression matrices).

### Correlations between controllability-driven behavioral, physiological and neuromolecular responses

The expression of all genes in the Dlv was positively correlated both with cortisol and with freezing behavior, and negatively correlated with exploratory behaviour (Table 2). A negative correlation was observed between cortisol and the expression of *bdnf* in the Vv. Finally, there were positive correlations between cortisol and freezing behavior and escape attempts (Table 2).

**Table 2:** Pearson correlations between controllability-driven behavioral, physiological and of IEGs mRNA responses expressed between experimental conditions (i.e. CTR = stressor controllability; UnCTR = stressor uncontrollability and CTRUn = loss of stressor controllability)

	Behaviours	Freezing behaviour			Escape Attempts			Exploratory Behaviour			Cortisol		
Brain nuclei	genes	n	Rp	p	n	Rp	p	n	Rp	p	n	Rp	p
DLV	egr1	24	0.483	0.017				24	− 0.465	0.022	23	0.444	0.034
	cfos	24	0.533	0.007				24	− 0.7	< 0.001	23	0.526	0.01
	bdnf	24	0.466	0.022				24	− 0.624	0.001	23	0.537	0.008
	npas4	24	0.492	0.015				24	− 0.498	0.013	23	0.535	0.008
VV	Bdnf										24	− 0.594	0.003
	Cortisol	69	0.473	< 0.001	69	0.426	< 0.001						

Time in freezing, frequency of escape events, and exploratory behavior expressed during the test session and cortisol levels and immediate early gene (IEGs) mRNA expression measured 30 min after the test session. IEGs mRNA from each of the candidate brain nuclei in seabass that presented significant correlations (i.e. Dlv and Vv) are indicated. Significant differences are expressed with * (p < 0.05)

### Gene expression and physiological state predict stress coping state

**Fig. 4:**
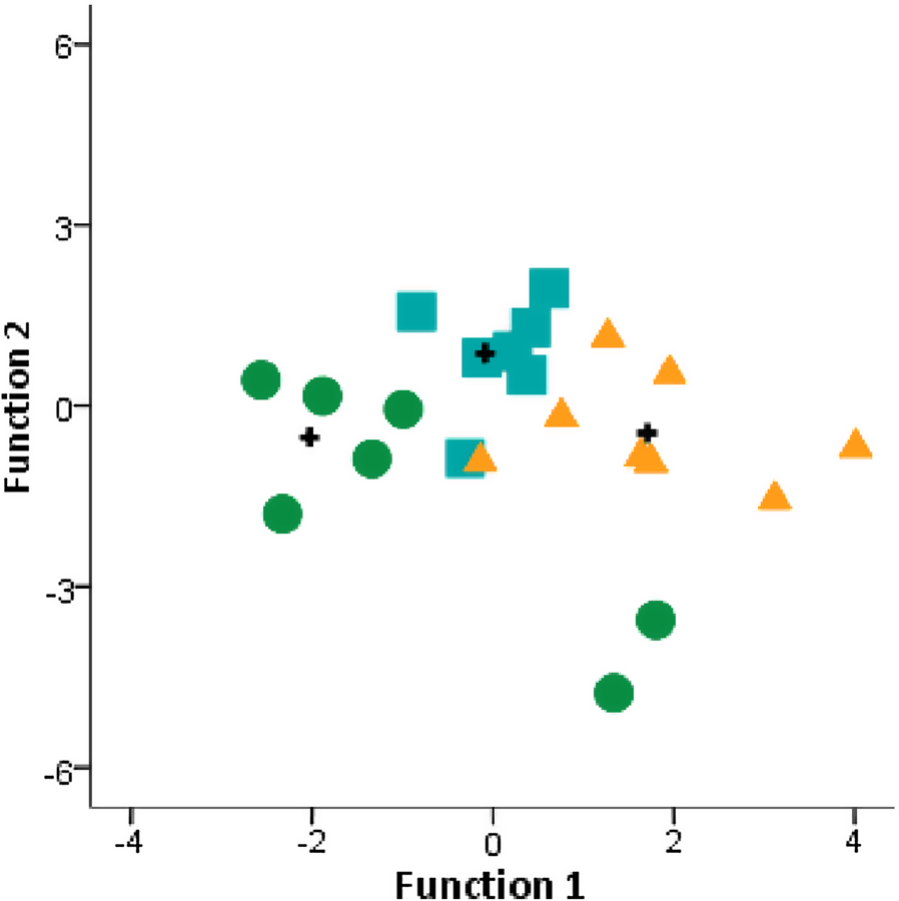
Linear discriminant analysis of cortisol and *egr-1*, *c-fos*, *bdnf* and *npas4* expressed in the candidate brain nuclei from seabass. The significant functions 1 and 2 highlight the three coping responses of fish under three experimental conditions: CTR = stressor controllability (circles); UnCTR = stressor uncontrollability; CTRUn = loss of stressor controllability (triangles). Discriminant scores for each individual are plotted and stars represent the centroid of each classified group

A linear discriminant function analysis, combining IEGs expression on the target brain nuclei and cortisol levels, was used to assess if the perception of controllability of the same stimuli elicits different internal states. Two discriminant functions were significantly loaded that explained 84% and 16% of variation (function 1: Wilk’s lambda = 0.171, chi-square = 18.57, p = 0.001; function 2: Wilk’s lambda = 0.651, chi-square = 4.50, p = 0.034; Fig. 4). Function 1 was significantly loaded by *bdnf* expression in Dld (0.691), whereas function 2 was significantly loaded by cortisol (0.786). These discriminant functions allowed the correct classification of 87.5% of either CTR or UnCTR and 75% of CTRUn (overall 83.3%). These 2 functions allowed the correct classification of all individuals according to their stress coping state.

## Discussion

In this study, we have shown that stressor controllability modulates the stress response of sea bass, as measured by behavioral, physiological and neural indicators. It is important to highlight that a conditioning protocol was used in this study and that in the test phase fish were responding to the CS alone and not to the stressor itself. Hence, the results presented herein confirm the findings of previous studies [43, 44], that during the test phase of the experiment sea bass have the ability to recall memories of previous aversive events that they have been exposed to in the training period. Given that the training period allowed individuals to assess their ability to control the stressor, their response in the test phase should reflect their stress-coping ability given the available information on stressor controllability.

At the behavioral level, the perception of control over the stressor significantly reduces the expression of defensive behaviors, namely freezing, escape attempts and shoal cohesion, and increases exploratory behavior. Furthermore, freezing, which is a universal fear response, characterized by a state of attentive immobility [45, 46], is higher in the group who lost control of the stressor than in the uncontrolled stressor treatment. These results are consistent with the finding in rodents that freezing is a hallmark of learned helplessness, lasting longer in situations where no escape from a stressor is available than when escaping is possible [1, 47]. Thus, it is plausible that sea bass have learned that outcomes in the loss of control treatment, came independent of their response, and hence further increased their freezing behavior. The higher shoal cohesion, observed in the uncontrollable and lack of control of stressor treatments, is in agreement with the use of shoal cohesion as a measure of anxiety and fear in fish [48, 49]. Thus, stressor controllability seems to reduce anxiety/fear in zebrafish facing an aversive stimulus.

At the physiological level, the response of circulating cortisol levels to the stressor was higher in the uncontrollable than in the controllable treatment, and the lack of control elicited even higher levels. Cortisol release is known to be involved in the modulation of arousal, vigilance, attention and memory formation [50], facilitating the encoding of emotion-related memory in fish [33]. Indeed, individuals in the controlled stressor treatment displayed the lowest cortisol concentrations, suggesting that the perception of control, rather than lack or loss of control over a stressful event, is effectively appraised as less harmful. Thus, and in accordance to previous research [37, 42, 51], psychological stress increases the synthesis and release of cortisol, in association with fear and anxiety states as suggested by the behavioral data. In fact, the cortisol results are in agreement with the behavioral data supporting a buffering role of controllability in the physiological stress response, and the occurrence of a helplessness state in lack or loss of control situations. Finally, it is worth noting that the cortisol levels measured in this study are within those previously reported for this species, even with different protocols [33, 43, 44].

At the neuromolecular level, the pattern of co-expression of the immediate early genes (*egr-1*, *c-fos*, and *npas4*) and the neural plasticity gene *bdnf*, used in this study to capture the neurogenomic state of target brain regions, was specific for each experimental treatment in each of the sampled brain regions (Dm, Dld, Dlv, Vv). Taking the expression of the immediate early genes as a proxy for neuronal activity, these results indicate that the perception of control, the lack of control and the loss of control of a stressor, are each represented at the brain level by a specific pattern of functional connectivity (i.e. pattern of co-activation) between these four brain regions. Finally, we have used linear discriminant function analyses to check if the measured behavioral, physiological and neuromolecular variables could efficiently discriminate the three experimental treatments. Two discriminant function correctly classified 83.3% of individuals according to the stressor coping state that they have been exposed to. Thus, physiological and neurogenomic state may well discriminate between three distinct stress coping states. Our data can be interpreted as evidence for the occurrence of cognitive abilities in fish corresponding to different appraisal of their environmental stressors.

Moreover, when brain regions are analyzed individually, they show different patterns.

There are no differences in the expression of any of the measured genes in Dm, the putative teleost homologue of the tetrapod pallial amygdala [28, 29], across the three experimental treatments, suggesting that this area is not involved in the processing of stress controllability. This is a surprising result, given the established role of Dm in emotional learning in teleost fish [27, 45] and the previous results in sea bass showing a differential expression of *egr1* in response to stressor predictability [41]. It is thus, possible that despite the fact that both predictability and controllability are cognitive appraisal variables that modulate the stress response they might be processed by different neural networks. In fact, while the former depends on stored information in memory about relations between stimuli (i.e. stimulus–stimulus learning or classical conditioning), the later depends on stored information about relations between responses and stimuli (i.e. stimulus–response learning or instrumental conditioning) [52]. Therefore, the effect of predictability is based on perceived contingencies between stimuli (i.e. stimulus expectancies), hence relying on the perceived probability of occurrence of the anticipated event; whereas controllability is based on contingencies between stimulus and response (i.e. response expectancies), hence relying on the perceived probability of response outcomes [33, 52]. Thus, whereas stimulus-stimulus contingencies seem to be associated with the differential activation of Dm, as measured by *egr1* expression, stimulus–response contingencies do not seem to require differential activation of this region.

In contrast, the four measured genes show similar differential patterns of expression in both in Dld and Dlv. In Dld, the teleost putative homologue of the sensory cortex [28, 29], the expression of all four genes was higher in the loss of control treatment than in the uncontrolled stressor treatment. Interestingly, the controlled stressor treatment elicited a pattern of gene expression for the four genes that is not significantly different from any of the other two treatments, but is much closer to the loss of control treatment. This suggests that the pattern of activation of this region reflects the commonalities between the controlled stressor and the loss of control treatment, which reside on the training during the first 5 training sessions in which similar contingencies between the CS stimulus and the escape response were established. Therefore, the activation of this area seems to be related to response expectancies. The fact that Dld is a sensory higher processing area that receives and integrates massive visual inputs [28, 29, 53], is congruent with the fact that the training used a visual CS. Also, the involvement of Dld in short-term memory and in the performance of activity under stressful situations in goldfish, suggests the potential involvement of this area in memory processes in fish [54]. In the Dlv, which is the putative teleost homologue of the hippocampus [28, 29], the expression of all four genes was significantly higher in the loss of control treatment than either in the controlled or uncontrolled stressor treatments, which have similar levels of expression. Therefore, the activation of this area seems to reflect the violation of expectancies in the test phase experienced by the individuals of the loss of control group, which may act as an error signal in the response expectancies. Although this region has been mainly associated with spatial learning, increasing evidence has linked its activity with time-related learning [30, 31], which might be the basis for its involvement in the here reported violation of response expectancies.

The Vv, which is the putative teleost homologue of the lateral septum and of the nucleus accumbens [34, 35], is the only brain region where the expression of the four genes was not similar, and only two genes showed different levels of expression between the experimental treatments, *egr-1* and *bdnf*. The expression of *egr-1* was higher in the uncontrollable stressor treatment than either in the controlled or loss of control treatment, which did not differ between them. Hence, this pattern of expression seems to be associated with the training for perceiving the stressor as uncontrollable (i.e. CS signals a lack of a coping response). It is important to note that *c-fos* and *npas1* have similar patterns, but the comparisons between treatments were not significant. Interestingly, these patterns of response are exactly the inverse of the patterns of expression of all four genes in the Dld reported above, which seem to be associated with the response expectancies (i.e. CS signals a coping escape response) in the controlled and loss of stressor control treatments. On the other hand, *bdnf* presents a specific pattern of expression in the Vv, with higher levels in controllable than in either uncontrollable or loss of stressor control treatments. This result is in line with the inhibition of *bdnf* mRNA expression in whole telencephalon samples in Atlantic salmon, in response to the omission of an expected reward [55]. Thus, the higher expression of *bdnf* in the Vv seems to be related to perceived stress coping which is probably associated with lower anxiety and fear. This interpretation is supported by the established role of *bdnf* in the nucleus accumbens on reward and motivational state [56].

The occurrence of stressor controllability in fish that lack the cortical areas (e.g. mPFC) known to regulate the stress response as a function of perceived stressor control, through a top-down inhibitory control over subcortical stress-responsive structures [15], illustrates how similar cognitive abilities (i.e. learning response contingencies) may be implemented by different neural circuits that have evolved separately in divergent vertebrate taxa (i.e. teleost vs. mammals). In recent years, research on the neuronal basis of the well develop cognitive capacities of birds, which also lack a layered cerebral cortex, reached similar conclusions. Irrespective of layered (in mammals) or nuclear (in birds) telencephalic neuronal architectures, cognitive ability seems to rely on associative capacities implemented by interneurons placed between sensory input and motor output neurons, which integrate sensory input and information stored in memory [23–25]. Thus, the view that fish are not able of complex cognitive abilities due to their relatively smaller brains that lack a cortical area homologous to that of mammals (e.g. [57, 58], which has direct implications for fish sentience and welfare, should be revised.

## Conclusions

Here we present behavioral, physiological and neuromolecular evidence for the modulation of the stress response by the perception of stressor controllability in fish, suggesting a deep evolutionary origin of this cognitive ability in vertebrate evolution, which is implemented by different neural substrates in divergent vertebrate taxa. These results also have implications for fish welfare opening the way for using psychological factors in the management of farmed fish, namely in handling and husbandry practices.

## Methods

### Experimental fish

Seabass were obtained from the experimental research station of IFREMER in Palavas-les-Flots (France) and housed in fibre glass tanks (500 L) during 8 months at Ramalhete Research Station from Centre of Marine Sciences (Faro, Portugal), until the start of the experiment in May of 2013. At the start of the experiments the body mass of the fish was 47.12 ± 6.80 g (mean ± SEM).

### Experimental procedures

Seventy-two individuals, randomly selected from the stock tank were measured, weighted and tagged with a floy tag (Floy Tag Manufacturing Inc, Seattle, USA) with a multicolour pearl attached behind the dorsal fin. Groups of 4 fish were randomly distributed by twelve experimental aquaria of approximately 80 l (70 × 40 × 30 cm depth) in an open water under standard housing conditions until the start of the experiments (temperature 18 ± 3 °C, salinity 35 ± 2 ‰, dissolved oxygen above 75%, and a 12 L: 12 D photoperiod). The experiment was developed in three runs, using 24 fish/run. All aquaria walls were covered with opaque partitions in order to prevent contact between focal animals and the experimenter. Each aquarium was provided with a confinement net attached to a white rigid structure, with the same size as the lateral walls of the tank (Additional file 1: Fig. S1). Each run lasted 12 days, including 8 days of acclimation to the experimental tanks and the last 4 to the experimental period (2 training sessions per day at 10:00 and 15:00 h; the last session of the fourth day was the test session). During this period fish were hand-fed twice a day at 08:00 h and 18:00 h at 3% of biomass day^−1^.

During the training sessions fish were trained to learn an association between a light (CS; on for 2 min) and a subsequent 5 min confinement (unconditioned stimulus: US). The light (12 V, 10 W) was hung in the top of a lateral wall of the aquarium, on the opposite side of the confinement net. Confinement consisted in moving the net into the light wall direction until reach 15% of the aquarium volume. Fish were tested under different conditions: i) Controllable situation (CTR)—fish had the choice to escape from US (confinement) by a door of 10 cm^2^; ii) Uncontrollable situation (UnCTR)—fish had no choice to escape from US; iii) Loss of Controllability (CTRUn)—fish were subjected during 5 conditioning sessions to the same conditions as CTR followed by 2 sessions under UnCTR conditions (Additional file 1: Table S2). In all cases, the event was created by signalling (CS) the aversive stimulus. In the test session all experimental groups were exposed to the CS in the absence of the US. Each aquarium was labelled with a different number to permit further blind analysis of the different samples taken.

### Behavioral analysis

Behavior was video recorded (top view) during the CS of the test session. Video recordings were labelled with a number by a researcher different from the one involved in the different analysis to permit a blind analysis of the behaviour. Behavioral responses of 72 fish to the CS was analysed with computerized multi-event recorder software (Observer XT®, from Noldus, Wageningen, Netherlands). The conditioned response was assessed by: time in freezing (total time without any movement), escape behavior (i.e. fish swimming strongly, going close to the tank walls or moving in a way consistent with escape attempts), shoal cohesion (1—low cohesion i.e., fish were spread in the tank; 2—medium cohesion i.e., 2 fish were together; 3—high cohesion i.e., three or more fish together) and exploratory behavior (measured by the time fish spent in different areas of the tank: 1—light side area; 2—centre of the tank; 3—confinement net area) summarized by the formula:

1 $${\text{A/t}}_{{{\text{maximum}}}}$$

where A is the arithmetic mean of the time fish spent in each one of the areas of the tank, and t the maximum time found for any of the areas tested. When exploratory behavior is high, this ratio should be close to 1, while it should be close to 0 when exploratory behavior is low.

### Blood collection and Hormone Analysis

Thirty min after the test session, fish were caught (n = 72) and euthanized with an overdose of 2-phenoxyethanol (1 ‰, Sigma-Aldrich). Blood was immediately collected from the caudal vein and centrifuged at 2000*g* for 25 min. Plasma was frozen in dry ice and stored at − 80 °C until further processing. Plasma cortisol were then determined using a commercial ELISA kit RE52061 (IBL Hamburg, Germany), with a sensitivity of 2.5 ng ml^−1^, and intra and inter-assay coefficients of variation (CV) of 2.9 and 3.5%, respectively. Samples were identified according to the videos labelling.

### Brain microdissection and immediate early genes (IEGs) expression

Eight individuals from each experimental treatment (n = 24) were randomly selected for the assessment of immediate early gene mRNA expression. After sacrifice (see above) the skull, with the brain inside, was removed from the fish, embedded in Tissue-Tek®, and kept at − 80 °C until further processing. Labelling of the samples were performed as for the blood samples. Thick brain telencephalon coronal Sects. (150 µm) were obtained using a cryostat (Leica, CM 3050S). Brain nuclei of interest (Dm, Dld, Dlv, and Vv), identified according to the available brain atlas [59, 60], were microdissected (see Additional file 1: Fig. S2 and Additional file 2 for detailed description). Tissue was collected directly into lysis buffer from Qiagen Lipid Tissue Mini Kit (#74804; Valencia, CA), total RNA extracted from the samples, reverse transcribed to cDNA (BioRad iScript cDNA Synthesis Kit; Valencia, CA), and used as a template for quantitative polymerase chain reactions (qPCR) of the following IEGs: *egr-1*, *c-fos*, *bdnf* and *npas4*. The geometric mean of the expression of two previously established housekeeping genes, *eef1a* and *18S*, was used as an internal control (see additional file for qPCR conditions and Additional file 1: Table S3 for primer sequences).

### Statistical analysis

Label scheme was deciphered before the statistical analysis to allocate each number to the respective treatment group. All samples were included in the analysis. The assumptions of normality and homoscedasticity were confirmed by analysis of the residuals whereas homogeneity of variance was checked by Levene’s test. Descriptive statistics are expressed as mean ± standard error of the mean (SEM) and log or arcsine transformation was used to achieve homogeneity when required.

A linear mixed model (LMM) was used to assess the effect of each experimental condition (i.e. CTR vs. UnCTR; CTR vs. CTRUn; UnCTR vs. CTRUn) on the behavioral variables assessed in the test session, on cortisol levels and on IEGs mRNA expression (*egr-1*, *c-fos*, *bdnf* and *npas4*) in each brain region of seabass (Dm, Dld, Dlv and Vv). Given that we have used more than one individual from the same tank in each condition, we accounted for sampling dependence by adding a random effect for the "tank" factor in each LMM. In general, we did not find an effect of the "tank" variable on the measured responses.

All LMM were estimated using the restricted maximum likelihood method. A priori planned comparisons were used to test for specific differences between experimental conditions, namely: CTR vs. UnCTR; CTR vs. CTRUn; UnCTR vs. CTRUn. Pearson correlations were used to depict the association between behavioral variables, between behavior and cortisol, and between those and gene expression.

Separate stepwise linear discriminant analyses (LDA) was used to define which brain nuclei state (i.e. immediate early genes expression in different brain nuclei) and cortisol expression better predict the coping responses to aversive events. The F test statistic was used as a measure of the contribution of each variable (cortisol concentration and IEGs expression in each brain region) to the discriminant functions. An F-value above 3.84 was used as the selection criteria for predictors to enter the model and predictors were removed when the F-value dropped below 2.71 (e.g. Maruska et al. [61]). Heatmaps of Pearson correlations matrices, with adjusted *p*-values [62] were used to assess the neurogenomic states of fish, where the quadratic assignment procedure (QAP) correlation test with 5000 permutations [63] test the differences in gene co-expression patterns between brain areas within each experimental condition, and between experimental conditions within each brain area. The null hypothesis of the QAP test is that when *p* > 0.05 there is no association between matrices, hence a non-significant p-value indicates that the correlation matrices are different. LMM, LDA, neurogenomic states and QAP correlations were performed using R® (R Development Core Team). Statistical significance was taken at *p* < 0.05.

## Supplementary Information

**Additional file 1.** Supplementary figures.

**Additional file 2.** Raw data.

## Data Availability

All data analyzed during this study are included in this published article and its supplementary information files.

## References

[1] Maier SF, Seligman MEP (2016). Learned helplessness at fifty: Insights from neuroscience. Psychol Rev.

[2] Brady JV (1958). Ulcers in executive monkeys. Sci Am.

[3] Seligman ME, Maier SF (1967). Failure to escape traumatic shock. J Exp Psychol.

[4] Maier SF, Seligman ME (1976). Learned helplessness: theory and evidence. J Exp Psychol Gen.

[5] Weiss JM (1968). Effects of coping responses on stress. J Comp Physiol Psychol.

[6] Weiss JM (1970). Somatic effects of predictable and unpredictable shock. Psychosom Med.

[7] Weiss JM (1971). Effects of coping behavior in different warning signal conditions on stress pathology in rats. J Comp Physiol Psychol.

[8] Hiroto DS, Seligman ME (1975). Generality of learned helplessness in man. J Pers Soc Psychol.

[9] Abramson LY, Seligman ME, Teasdale JD (1978). Learned helplessness in humans: Critique and reformulation. J Abnorm Psychol.

[10] Alloy LB, Peterson C, Abramson LY, Seligman ME (1984). Attributional style and the generality of learned helplessness. J Pers Soc Psychol.

[11] Hartley CA, Gorun A, Reddan MC, Ramirez F, Phelps EA (2014). Stressor controllability modulates fear extinction in humans. Neurobiol Learn Mem.

[12] Henderson RK, Snyder HR, Gupta T, Banich MT (2012). When does stress help or harm? The effects of stress controllability and subjective stress response on stroop performance. Front Psychol.

[13] Markus R, Panhuysen G, Tuiten A, Koppeschaar H (2000). Effects of food on cortisol and mood in vulnerable subjects under controllable and uncontrollable stress. Physiol Behav.

[14] Müller MJ (2011). Helplessness and perceived pain intensity: relations to cortisol concentrations after electrocutaneous stimulation in healthy young men. BioPsychoSocial medicine.

[15] Maier SF (2015). Behavioral control blunts reactions to contemporaneous and future adverse events: medial prefrontal cortex plasticity and a corticostriatal network. Neurobiol Stress.

[16] Kern S (2008). Glucose metabolic changes in the prefrontal cortex are associated with HPA axis response to a psychosocial stressor. Psychoneuroendocrinology.

[17] Sugaya N (2012). Adrenal hormone response and psychophysiological correlates under psychosocial stress in individuals with irritable bowel syndrome. Int J Psychophysiol.

[18] Maier SF, Ryan SM, Barksdale CM, Kalin NH (1986). Stressor controllability and the pituitary-adrenal system. Behav Neurosci.

[19] Helmreich DL (1999). The effect of stressor controllability on stress-induced neuropeptide mRNA expression within the paraventricular nucleus of the hypothalamus. J Neuroendocrinol.

[20] Sanchís-Ollé M (2019). Controllability affects endocrine response of adolescent male rats to stress as well as impulsivity and behavioral flexibility during adulthood. Sci Rep.

[21] Venkatesh B, Erdmann MV, Brenner S (2001). Molecular synapomorphies resolve evolutionary relationships of extant jawed vertebrates. Proc Natl Acad Sci.

[22] Ito H, Yamamoto N (2009). Non-laminar cerebral cortex in teleost fishes?. Biol Lett.

[23] Briscoe SD, Ragsdale CW (2019). Evolution of the Chordate Telencephalon. Curr Biol.

[24] Mehlhorn J, Hunt GR, Gray RD, Rehkämper G, Güntürkün O (2010). Tool-making new caledonian crows have large associative brain areas. Brain Behav Evol.

[25] Olkowicz S (2016). Birds have primate-like numbers of neurons in the forebrain. Proc Natl Acad Sci.

[26] Lal P (2018). Identification of a neuronal population in the telencephalon essential for fear conditioning in zebrafish. BMC Biol.

[27] Portavella M, Torres B, Salas C (2004). Avoidance response in goldfish: emotional and temporal involvement of medial and lateral telencephalic pallium. J Neurosci.

[28] Demski LS (2013). The pallium and mind/behavior relationships in teleost fishes. Brain Behav Evol.

[29] Ganz J (2014). Subdivisions of the adult zebrafish pallium based on molecular marker analysis. F1000 Research.

[30] Ocaña FM, Uceda S, Arias JL, Salas C, Rodríguez F (2017). Dynamics of goldfish subregional hippocampal pallium activity throughout spatial memory formation. Brain Behav Evol.

[31] Rodríguez F, et al. Conservation of spatial memory function in the pallial forebrain of reptiles and ray-finned fishes. J Neurosci. 2002;22: 2894. 10.1523/JNEUROSCI.22-07-02894.2002 PMC6758289 11923454

[32] Uceda S (2015). Spatial learning-related changes in metabolic brain activity contribute to the delimitation of the hippocampal pallium in goldfish. Behav Brain Res.

[33] Cerqueira M (2017). Cognitive appraisal of environmental stimuli induces emotion-like states in fish. Sci Rep.

[34] Ganz J (2012). Subdivisions of the adult zebrafish subpallium by molecular marker analysis. J Comp Neurol.

[35] Goodson JL, Kingsbury MA (2013). What's in a name? Considerations of homologies and nomenclature for vertebrate social behavior networks. Horm Behav.

[36] Galhardo L, Oliveira RF (2009). Psychological Stress and Welfare in Fish. ARBS Ann Rev Biomed Sci.

[37] Galhardo L, Vital J, Oliveira RF (2011). The role of predictability in the stress response of a cichlid fish. Physiol Behav.

[38] Madaro A (2016). Effect of predictability on the stress response to chasing in Atlantic salmon (*Salmo salar* L.) parr. Physiol Beh.

[39] Madaro A (2015). Stress in Atlantic salmon: response to unpredictable chronic stress. J Exp Biol.

[40] Vindas MA (2014). Frustrative reward omission increases aggressive behavior of inferior fighters. Proc R Soc B.

[41] Cerqueira M (2020). Cognitive appraisal in fish: stressor predictability modulates the physiological and neurobehavioral stress response in sea bass. Proc R Soc B.

[42] Carpenter RE, Summers CH (2009). Learning strategies during fear conditioning. Neurobiol Learn Mem.

[43] Millot S (2014). Use of conditioned place preference/avoidance tests to assess affective states in fish. Appl Anim Beh Sci.

[44] Millot S (2014). Behavioral stress responses predict environmental perception in european sea bass dicentrarchus labrax. PLoS ONE.

[45] Lang PJ, Davis M (2006). Emotion, motivation, and the brain: reflex foundations in animal and human research. Prog Brain Res.

[46] Lojowska M, Gladwin TE, Hermans EJ, Roelofs K (2015). Freezing promotes perception of coarse visual features. J Exp Psychol Gen.

[47] Blanchard DC, Griebel G, Pobbe R, Blanchard RJ (2011). Risk assessment as an evolved threat detection and analysis process. Neurosci Biobehav Rev.

[48] Gerlai R (2010). Zebrafish antipredatory responses: a future for translational research?. Behav Brain Res.

[49] Stewart A (2012). Modeling anxiety using adult zebrafish: a conceptual review. Neuropharmacology.

[50] van Ast VA (2013). Modulatory mechanisms of cortisol effects on emotional learning and memory: Novel perspectives. Psychoneuroendocrinology.

[51] Øverli Ø (2004). Behavioral and neuroendocrine correlates of displaced aggression in trout. Horm Behav.

[52] Ursin H, Eriksen HR (2004). The cognitive activation theory of stress. Psychoneuroendocrinology.

[53] Wullimann MF, Mueller T (2004). Teleostean and mammalian forebrains contrasted: evidence from genes to behavior. J Comp Neurol.

[54] Vargas JP, López JC, Portavella M (2009). What are the functions of fish brain pallium?. Brain Res Bull.

[55] Vindas MA (2014). Coping with Unpredictability: Dopaminergic and Neurotrophic Responses to Omission of Expected Reward in Atlantic Salmon (*Salmo salar* L.). PLoS ONE.

[56] Koo JW, Chaudhury D, Han M-H, Nestler EJ (2019). Role of Mesolimbic Brain-Derived Neurotrophic Factor in Depression. Biol Psychiat.

[57] Rose JD (2002). The neurobehavioral nature of fishes and the question of awareness and pain. Rev Fish Sci.

[58] Rose JD (2014). Can fish really feel pain?. Fish Fish.

[59] Cerda-Reverter JM, Zanuy S, Munoz-Cueto JA (2001). Cytoarchitectonic study of the brain of a perciform species, the sea bass (Dicentrarchus labrax) I The telencephalon. J Morphol.

[60] Munoz-Cueto JA, Sarasquete C, Zohar AH, Kah O. An atlas of the brain of the gilthead seabream (Sparus aurata). *College Park: Maryland Sea Grant*; 2001.

[61] Maruska KP, Zhang A, Neboori A, Fernald RD (2013). Social opportunity causes rapid transcriptional changes in the social behavior network of the brain in an African cichlid fish. J Neuroendocrinol.

[62] Benjamini Y, Hochberg Y (1995). Controlling the false discovery rate: a practical and powerful approach to multiple testing. J R Stat Soc.

[63] Borgatti SP, Everett MG, Johnson JC (2013). Analyzing social networks.

