# Supplementary material for: Stressor controllability modulates the stress response in fish
Source: BMC Neurosci. 2021 Aug 4;22:48. doi: 10.1186/s12868-021-00653-0 (PMC8336412; doi:10.1186/s12868-021-00653-0)
Supplement: Supplementary file 1 — Additional file 1. Supplementary figures. [file 12868_2021_653_MOESM1_ESM.docx]

**Stressor controllability modulates the stress response in fish**

M. Cerqueira, S. Millot, T. Silva, A.S. Félix, M.F. Castanheira, C.I.M. Martins, S. Rey, S. MacKenzie, R.F. Oliveira, C.C.V. Oliveira.

Correspondence to:

Rui F. Oliveira,

Integrative Behavioural Biology

Instituto Gulbenkian de Ciência,

Rua da Quinta Grande 6,

2780-156 Oeiras, Portugal

ruiol@ispa.pt

**Table S1** Quadratic assignment procedure (QAP) correlation test for the different brain nuclei. Dm, medial zone of the dorsal telencephalic area; Dld, dorsal lateral zone of the dorsal telencephalic area; Dlv, ventral lateral zone of the dorsal telencephalic area; Vv, ventral nucleus of the ventral telencephalic area between experimental conditions CTR = controllability; UnCTR = controllability; CTRUn = loss of controllability; Significant correlations are indicated in bold for p < 0.05.

|  |  | **Brain nuclei** | | | |
| --- | --- | --- | --- | --- | --- |
| **Experimental conditions** |  | **Dm** | **Dld** | **Dlv** | **Vv** |
| CTR-UnCTR | *r* | 0,576 | 0,315 | -0,626 | -0,35 |
|  | *p* | 0,214 | 0,293 | 0,09 | **0,046** |
| CTR-CTRUn | *r* | -0,322 | 0,172 | 0,188 | 0,535 |
|  | *p* | 0,371 | 0,341 | 0,301 | 0,203 |
| UnCTR-CTRUn | *r* | 0,465 | 0,04 | -0,391 | 0,076 |
|  | *p* | 0,259 | 0,505 | 0,334 | 0,452 |
|  |  |  |  |  |  |
|  |  | **Experimental conditions** | | |  |
| **Brain nuclei** |  | **CTR** | **UnCTR** | **CTRUn** |  |
| Dm-Dld | *r* | 0,398 | 0,476 | 0,546 |  |
|  | *p* | 0,305 | **0,041** | 0,172 |  |
| Dm-Dlv | *r* | 0,966 | 0,242 | -0,203 |  |
|  | *p* | **0,042** | 0,466 | 0,414 |  |
| Dm-Vv | *r* | 0,169 | 0,045 | -0,232 |  |
|  | *p* | 0,297 | 0,373 | 0,292 |  |
| Dld-Dlv | *r* | 0,457 | 0,354 | -0,664 |  |
|  | *p* | 0,293 | 0,13 | **0,046** |  |
| Dld-Vv | *r* | 0,958 | -0,074 | 0,041 |  |
|  | *p* | **0,044** | 0,42 | 0,426 |  |
| Dlv-Vv | *r* | 0,203 | 0,501 | 0,411 |  |
|  | *p* | 0,286 | 0,252 | 0,246 |  |



**Figure S1** Experimental set-up used in the research attending stress coping ability of fish under controllable (CTR), uncontrollable UnCTR) and loss of control conditions CTRUn)

**Table S2** Training procedures for each experimental condition tested for seabass

| **Trial** | **Valence** | **Tanks** | **Possibility to escape** | **Training sessions**  **(1^st^ to 7^th^ session)** | **Test session**  **(8^th^ session)** |
| --- | --- | --- | --- | --- | --- |
| **Controllable** | Aversive n=48 | 6/each species | Yes | 1min CS + 1min CS overlapped with 1 min USavr + 4 min only USavr | 1 min CS |
| **CTR** |  |  | = 7 sessions |  |  |
| **Uncontrollable** |  |  | No |  |  |
| **UnCTR** |  |  | = 7 sessions |  |  |
| **Loss of Controllability CTRUn** |  |  | Yes |  |  |
|  |  |  | = 1^st^ to 5^th^ sessions |  |  |
|  |  |  | No |  |  |
|  |  |  | = 6^th^ and 7^th^ session |  |  |

**Brain microdissection and gene expression analysis**

Eight individuals of each experimental condition were quickly decapitated by cervical transection and the whole skull (with the brain) removed, immediately embedded in Tissue-Tek® and kept at -80 ºC until further processing. Brains were sliced through 150 µm thick cryostat (Leica, CM 3050S) coronal sections, put onto regular glass slices pre-cleaned with 70% ethanol, and microdissected with modified 25G steel needles. Three nuclei of interest were selected accordingly to homologies between the fish and mammals’ brain (1, 2) and identified accordingly to the brain atlas of this species (3) i.e. Dm, medial zone of the dorsal telencephalic area (basolateral amygdala), Dl, lateral telencephalic area (hippocampus) and ventral nucleus of the ventral telencephalic area (septum). The Dl was divided in both dorsal (Dld) and ventral divisions (Dlv); (see detailed description in Fig. S2). Tissue was collected directly into lysis buffer from Qiagen Lipid Tissue Mini Kit (#74804; Valencia, CA) and stored at -80 ºC until further processing (see below).

**RNA extraction (adapted from previous works developed in our laboratory)**

Tissue was homogenized in quiazol lysis reagent by vortex followed by an incubation of 7 min at room temperature (RT). It was added chloroform in a proportion of 1:2 and the sample incubated at RT for 5 min. Samples were then centrifuge at 13000 g for 20 min at 4 ºC, and the upper aqueous phase transferred to new tube where 1 volume of 70 % ethanol was added. This mixture was transferred to an RNEasy column, remained 5 min at RT, being subsequently centrifuged for 1 min at 9000 g. A sequence of buffers was added to the RNEasy column according to the manufacturer’s instructions, and RNA eluted with 25 µl of RNAse-free water. Each sample was then taken to Nanodrop to assess the “quality” of the RNA.

**Primers design and quantitative real-time PCR (qPCR)**

The qRT-PCR protocol was based and adapted from procedures previously performed by Desjardins and Fernald (4). Partial sequences for 18S (accession # AM490061.1), eef1a (accession # AF184170.1), egr-1 (accession # KC442101.1) and c-fos (accession #GU108576.1), were withdrawn from the National Center for Biotechnology Information (NCBI, <http://www.ncbi.nlm.nih.gov/nuccore>). For bdnf and npas4, primers were designed using NCBI sequences from several fish species and then aligned with ClustalW to select the most conserved regions (www.genome.jp/tools/clustalw) (5). Primers for all target mRNA were designed using Primer3 software (6, 7) and synthetized by Sigma-Aldrich (Hamburgo, German). The PCR products were sequenced to confirm the desired primer cDNA amplification. Primer dimmers formation was controlled with FastPCR v5.4 software (8, 9) and optimal annealing temperature was assessed for maximal fluorescence (Table S2 and S3). The qRT-PCR carried out using a Roche Light Cycler 480 II (Roche Diagnostics, Penzberg, Germany), was performed using 25 µL reactions including 12.5 µL Light cycler H 480 SYBR Green I Master (Roche diagnostics GmbH, Mannheim, Germany), 0.2 µL of each primer and 1 µL of cDNA template (RNA equivalent). Cycling conditions were as follows: (i) denaturation (5 min at 95 °C); (ii) amplification and quantification (40 cycles; 30 s at 95 °C, 45 s at primer specific annealing temperature (see table S2 and S3 for details), 30 s at 72 °C with a single fluorescence measurement); and (iii) melting curve assessment (30 s at 95 °C; 30 s at 55 °C, followed by an 55–95 °C with a heating rate of 0.5 °C/s and a continuous fluorescence measurement; 30 s at 95 °C).


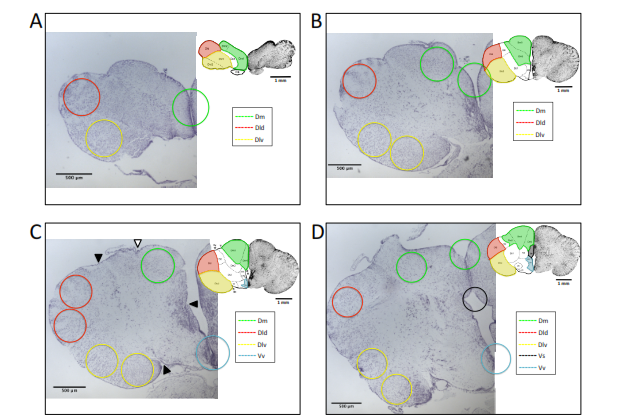


**Fig. S2** Coronal sections of Atlantic seabass (*Dicentrarchus labrax*) telencephalon spaced 400 µm from each other. Nissl staining images and an illustrative section adapted from Cerda-Reverter, et al. [13] highlighting the areas of interest from the telencephalon. Areas of interest: medial part of the dorsal telencephalon (Dm), lateral dorsal telencephalon (Dld, red), lateral ventral telencephalon (Dlv, yellow), ventral nucleus of the ventral telencephalon (Vv, blue).

**Table S3** List of primers and respective temperature of annealing used for quantitative real-time PCR from the different telencephalon regions studied in Atlantic seabass (*Dicentrarchus labrax*) brain.

| **Gene** | **Forward Primer** | **Reverse Primer** | **Ta*** |
| --- | --- | --- | --- |
| **18S^1^** | ATGCGTGCATTTATCAGACC | CGAAAGTTGATAGGGCAGACA | 58 |
| **EeF1a^1^** | TGGCTTCAACATCAAGAACG | ATGTGAGCTGTGTGGCAATC | 57 |
| **c-Fos^2^** | GCCTGCACCACCTTTACTTC | AGAGGACTGGTCGTTGCTGT | 59 |
| **EGR-1^2^** | GCAGAAGGACAAGAAAGCAGA | GGGGTAAGAAGACACTGGAGA | 58 |
| **BDNF^2^** | GCTCAGCGTGTGTGACAGTA | ACAGGGACCTTTTCCATGAC | 57 |
| **NPAS4^2^** | CAACCAAAGGAGCATCCAAG | AGCCGTGCTTTATCTGCATC | 57 |

*Ta = Temperature of annealing; ^1^Reference genes; ^2^Target genes.

**References**

1. O'Connell LA & Hofmann HA (2011) The vertebrate mesolimbic reward system and social behavior network: a comparative synthesis. *J Comp Neurol* 519(18):3599-3639.

2. Goodson JL & Kingsbury MA (2013) What's in a name? Considerations of homologies and nomenclature for vertebrate social behavior networks. *Hormones and behavior* 64(1):103-112.

3. Cerda-Reverter JM, Zanuy S, & Munoz-Cueto JA (2001) Cytoarchitectonic study of the brain of a perciform species, the sea bass (Dicentrarchus labrax). I. The telencephalon. *J Morphol* 247(3):217-228.

4. Desjardins JK & Fernald RD (2010) What do fish make of mirror images? *Biology Letters* 6(6):744-747.

5. Thompson JD, Higgins DG, & Gibson TJ (1994) CLUSTAL W: improving the sensitivity of progressive multiple sequence alignment through sequence weighting, position-specific gap penalties and weight matrix choice. *Nucleic Acids Res* 22(22):4673-4680.

6. Koressaar T & Remm M (2007) Enhancements and modifications of primer design program Primer3. *Bioinformatics (Oxford, England)* 23(10):1289-1291.

7. Untergasser A*, et al.* (2012) Primer3--new capabilities and interfaces. *Nucleic Acids Res* 40(15):e115.

8. Kalendar R, Lee D, & Schulman A (2011) Java web tools for PCR, in silico PCR, and oligonucleotide assembly and analysis *Genomics* 98((2)):137-144.

9. Kalendar R, Lee D, & Schulman A (2014) FastPCR Software for PCR, In Silico PCR, and Oligonucleotide Assembly and Analysis. *DNA Cloning and Assembly Methods,* Methods in Molecular Biology, eds Valla S & Lale R (Humana Press), Vol 1116, pp 271-302.
